# Cerebral Microbleeds on Magnetic Resonance Imaging and Anticoagulant-Associated Intracerebral Hemorrhage Risk

**DOI:** 10.3389/fneur.2012.00133

**Published:** 2012-09-19

**Authors:** Andreas Charidimou, Clare Shakeshaft, David J. Werring

**Affiliations:** ^1^Stroke Research Group, Department of Brain Repair and Rehabilitation, The National Hospital for Neurology and Neurosurgery, UCL Institute of NeurologyQueen Square, London, UK

**Keywords:** cerebral microbleeds, cerebral small vessel disease, cerebral amyloid angiopathy, intracerebral hemorrhage, atrial fibrillation, anticoagulation, antithrombotics

## Abstract

The increasing use of antithrombotic drugs in an aging population [including anticoagulants to prevent future ischemic stroke in individuals with atrial fibrillation (AF)] has been associated with a dramatic increase in the incidence of intracerebral hemorrhage (ICH) in users of antithrombotic drugs. Several lines of evidence suggest that cerebral small vessel disease (particularly sporadic cerebral amyloid angiopathy) is a risk factor for this rare but devastating complication of these commonly used treatments. Cerebral microbleeds (CMBs) have emerged as a key MRI marker of small vessel disease and a potentially powerful marker of future ICH risk, but adequately powered, high quality prospective studies of CMBs and ICH risk on anticoagulation are not available. Further data are urgently needed to determine how neuroimaging and other biomarkers may contribute to individualized risk prediction to make anticoagulation as safe and effective as possible. In this review we discuss the available evidence on cerebral small vessel disease and CMBs in the context of antithrombotic treatments, especially regarding their role as a predictor of future ICH risk after ischemic stroke, where risk-benefit judgments can be a major challenge for physicians. We will focus on patients with AF because these are frequently treated with anticoagulation. We briefly describe the rationale and design of a new prospective observational inception cohort study (Clinical Relevance of Microbleeds in Stroke; CROMIS-2) which investigates the value of MRI markers of small vessel disease (including CMBs) and genetic factors in assessing the risk of oral anticoagulation-associated ICH.

## Introduction and Scope

Over the last decade, increasing use of oral anticoagulants to prevent cardioembolic stroke due to atrial fibrillation (AF) in an aging population, has been associated with a fivefold increase in the incidence of anticoagulation-associated intracerebral hemorrhage (ICH; Flaherty et al., 2007) – a rare, but unpredictable and catastrophic complication. Magnetic resonance imaging (MRI) can identify the presence and severity of cerebral small vessel diseases, including cerebral amyloid angiopathy (CAA) and hypertensive arteriopathy, which may predispose to ICH in elderly patients treated with antithrombotic agents (Pantoni, 2010). Key MRI markers of small vessel disease include cerebral microbleeds (CMBs) on T2*-weighted gradient-recalled echo (T2*-GRE) MRI (Greenberg et al., 2009; Charidimou and Werring, 2011) and leukoaraiosis (also known as white matter changes). Some studies suggest that leukoaraiosis increases the risk of oral anticoagulant-associated ICH, but the predictive value is modest (Pantoni, 2010). CMBs are a more recently recognized imaging finding, which provides direct evidence of leakage of blood from pathologically fragile small vessels (Charidimou and Werring, 2011), so they may logically be a stronger predictor of anticoagulant-associated ICH.

Several lines of evidence support the hypothesis that CMB distribution in the brain reflects the underlying small vessel disease: strictly lobar CMBs may relate to CAA, whilst deep CMBs likely reflect hypertensive arteriopathy (Greenberg et al., 2009; Pantoni, 2010; Charidimou and Werring, 2011). The risk of recurrent bleeding after symptomatic ICH seems to be higher for lobar ICH (often presumed due to CAA; Vinters, 1987; Passero et al., 1995). Lobar CMBs, suggesting CAA may thus be a stronger risk factor for antithrombotic-associated ICH than deep CMBs, but definitive data are lacking. A small prospective study showed that aspirin might be associated with recurrent lobar ICH in patients with CAA (Biffi et al., 2010a).

In this review (Table 1) we discuss the available evidence on CMBs in the context of antithrombotic treatments, especially regarding their role as a predictor of future ICH risk after ischemic stroke. We focus on patients anticoagulated after ischemic stroke associated with AF, and describe the methods and rationale of a new prospective observational inception cohort study (CROMIS-2^1^) investigating the value of MRI markers of small vessel disease (including CMBs) and genetic factors, in assessing the risk of oral anticoagulation-associated ICH.

**Table 1:** **Search strategy and selection criteria**.

References for this review were identified through PubMed (between January 1990, to April 2012) using the search terms: (a) “microbleed(s),” or “micro(-)h(a)emorrhage(s),” or “petechial h(a)emorrhage(s),” or “gradient-echo,” “T2*,” or “susceptibility,” and (b) “atrial fibrillation,” “anticoagulants,” “warfarin,” and “dabigatran.” The reference list from retrieved articles, related review articles, clinical guidelines and the authors’ own files were also searched for relevant publications. Searches focused on English-language sources and studies in human subjects. The final reference list was chosen on the basis of relevance to the topics covered in this article.

## Atrial Fibrillation, Oral Anticoagulation, and Anticoagulation-Associated ICH

Atrial fibrillation is the most common sustained cardiac rhythm disorder and it is increasing in prevalence and incidence because of an aging population (Miyasaka et al., 2006; Lip et al., 2012). The lifetime risk for developing AF is about one in four for men and women over the age of 40 (Lloyd-Jones et al., 2004). Untreated, AF increases the risk of ischemic stroke fivefold – making stroke the leading complication of AF (Lip et al., 2012). However, this risk of ischemic stroke increases with the presence of other stroke risk factors (Hughes and Lip, 2008).

Prevention of ischemic stroke is thus a major therapeutic goal in AF (Camm et al., 2010), for which oral anticoagulation is very effective in reducing risk by about 65%. This benefit has to be balanced against an increased risk of ICH, the most feared complication of oral anticoagulation which causes death or severe disability in up to 75% of patients (Fang et al., 2007). The careful assessment of ischemic stroke risk versus anticoagulation-related ICH risk by physicians is difficult, since paradoxically many of the known factors that increase ischemic stroke risk overlap with bleeding risk factors in patients with AF (Lip et al., 2011). This is illustrated in the current schemes for the assessment of ischemic stroke (CHA2DS2-VASc) and bleeding risk (HAS-BLED) advocated in the new European guidelines for the management of AF (Table 2; Camm et al., 2010): prior stroke or TIA and older age (≥75 years), are strong risk factors for both ischemic stroke and anticoagulation-associated ICH in patients with AF.

**Table 2:** **Current risk stratification schemes advocated in the new European guidelines for the management of atrial fibrillation (Camm et al., 2010) for the assessment of ischemic stroke (CHA2DS2-VASc) and bleeding risk (HAS-BLED) in patients with atrial fibrillation**.

	Score
**CHA_2_DS_2_-VASc**	
Congestive heart failure	1
Hypertension	1
Age ≥75 years	2
Diabetes mellitus	1
Stroke, TIA, or thromboembolism	2
Vascular disease^a^	1
Age 65–74 years	1
Sex category (i.e., female sex)	1
Maximum score	9
**HAS-BLED^b^**	
Hypertension (systolic blood pressure > 160 mm Hg)	1
Abnormal renal and liver function (1 point each)	1 or 2
Stroke	1
Bleeding tendency or predisposition	1
Labile international normalized ratios (if on warfarin)	1
Elderly (e.g., age >65 years)	1
Drugs or alcohol (1 point each)	1 or 2
Maximum score	9

*^a^Previous myocardial infarction, peripheral artery disease, or aortic plaque*.

*^b^“Hypertension” is defined as systolic blood pressure 160 mmHg. “Abnormal kidney function” is defined as the presence of chronic dialysis or renal transplantation or serum creatinine ≥200 mmol/L. “Abnormal liver function” is defined as chronic hepatic disease (e.g., cirrhosis) or biochemical evidence of significant hepatic derangement (e.g., bilirubin.2× upper limit of normal, in association with aspartate aminotransferase/alanine aminotransferase/alkaline phosphatase.3× upper limit normal, etc.). “Bleeding” refers to previous bleeding history and/or predisposition to bleeding, e.g., bleeding diathesis, anemia, etc. “Labile INRs” refers to unstable/high INRs or poor time in therapeutic range (e.g., 60%). Drugs/alcohol use refers to concomitant use of drugs, such as antiplatelet agents, non-steroidal anti-inflammatory drugs, or alcohol abuse, etc. INR 1/4 international normalized ratio. Adapted from Pisters et al. (2010)*.

*TIA, transient ischemic attack*.

**Figure 1:**
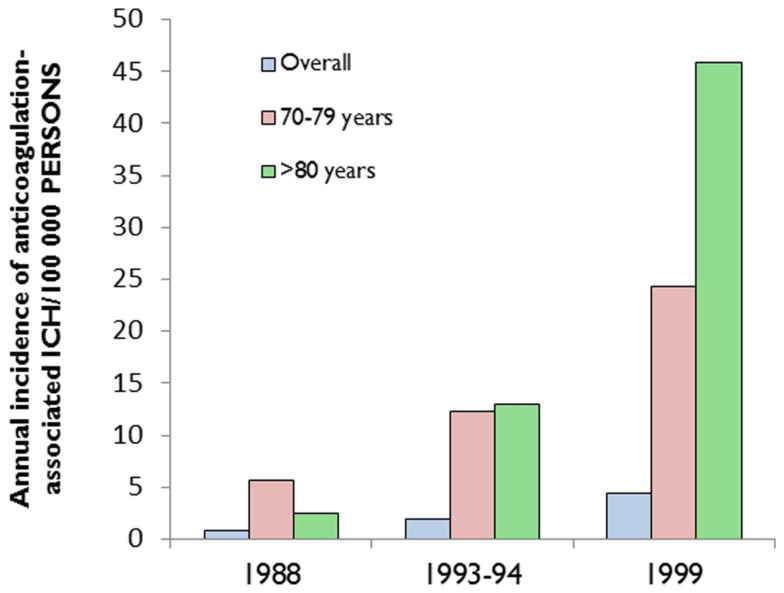
**The increasing incidence of anticoagulation-associated intracerebral hemorrhage (ICH), especially in the elderly**. Data extracted from (Flaherty et al., 2007).

A hospital-based study from the Greater Cincinnati area showed that the percentage of ICH associated with anticoagulant use increased from 5% in 1988 to 17% in 1999 (Figure 1; Flaherty et al., 2007). More recent studies from other areas have revealed similar trends (Kucher et al., 2004; Lovelock et al., 2007). Population-based data from Oxfordshire, UK (between 1981 and 2006) showed that the incidence of ICH associated with anticoagulant use has substantially increased (rate ratio: 7.4, 95% CI: 1.7–32; *p* = 0·007) among patients aged 75 years or older, and that while the incidence of deep (probably hypertensive arteriopathy-related) ICH has fallen, the proportion of non-hypertensive lobar bleeds in those aged 75 years or over increased (odds ratio 4.0, 95% CI: 1.1–17.4; *p* = 0.03; Lovelock et al., 2007). It is likely that CAA is implicated in the majority of these lobar hemorrhages and might also account for the increased incidence of anticoagulation-associated ICH (see below; Charidimou et al., 2012).

### Absolute risk of ICH in patients taking anticoagulants for atrial fibrillation: RCTs versus “real life” practice

**Figure 2:**
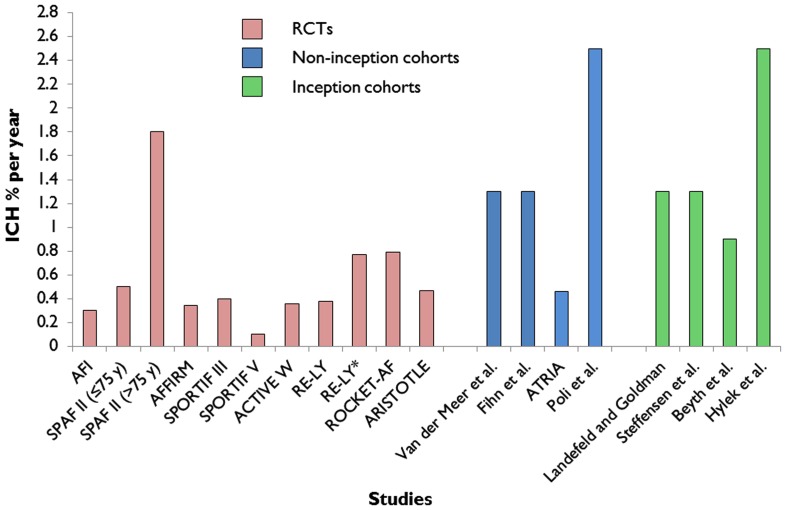
**Extracted data on reported annual rates of warfarin-associated ICH from major randomized controlled trials (RCTs; Atrial-Fibrillation-Investigators, 1994; SPAF-Investigators, 1994; Olsson, 2003; Albers et al., 2005; DiMarco et al., 2005; Connolly et al., 2006, 2009; Diener et al., 2010; Granger et al., 2011; Patel et al., 2011) of atrial fibrillation compared to non-inception cohort (Fihn et al., 1993; van der Meer et al., 1993; Go et al., 2003; Poli et al., 2009) and inception cohort studies (Landefeld and Goldman, 1989; Steffensen et al., 1997; Beyth et al., 1998; Hylek et al., 2007)**. *Subgroup analysis of patients with a previous history of ischemic stroke or transient ischemic attack in the RE-LY trial.

The absolute risk of ICH in an individual patient with AF taking anticoagulants is very difficult to determine from the results of published clinical studies: the reported rates of ICH vary widely ranging from 0.1% to more than 2.5% per year (Figure 2; Lip et al., 2011). These different rates are largely due to the heterogeneity of patient characteristics included in the studies (including the proportions with prior stroke and anticoagulant use).

Evidence-based management strategies based on whether a treatment works (and has its desired effect) are ideally based on randomized controlled trials (RCTs). However, RCTs designed to evaluate the efficacy of warfarin in AF may not be the optimum design to investigate rare adverse events (Vandenbroucke, 2011). Randomized trial participants are likely to be selected by physicians as “good candidates” based on a lower perceived bleeding risk profile and higher likelihood of adherence, or cases in whom physicians were uncertain as to best treatment, which will reduce the likelihood of serious adverse events. Indeed, many of the factors that that are known to increase the risk of bleeding are exclusion criteria in many RCTs of anticoagulation in AF. In six key trials that demonstrated the superiority of warfarin over placebo in the prevention of thromboembolic complications in AF, 28,787 patients were screened, but only 12.6% of these were included in the studies (Levi and Hovingh, 2008). The proportion of patients who are new to warfarin (“warfarin-naïve” patients) can also modify the results of RCTs, since the risk of both anticoagulation-associated ICH and ischemic stroke are highest in newly diagnosed patients with AF and during the first initiation of anticoagulant medication (Garcia et al., 2010).

Thus, the results of RCTs may not generalize to the “real world” patient population, and could underestimate the risk of important adverse events including ICH. Indeed, there is evidence and theory suggesting that observational studies are most likely to give correct estimates of the risk of serious adverse events, which are unintended and often unpredictable. Observational cohort studies are likely to have much higher rates of serious adverse effects than the highly selected populations included in RCTs, which makes this design a powerful way to assess predictors of risk (Vandenbroucke, 2011).

The reported annual rates of warfarin-associated ICH from major RCTs of AF (Atrial-Fibrillation-Investigators, 1994; SPAF-Investigators, 1994; Olsson, 2003; Albers et al., 2005; DiMarco et al., 2005; Connolly et al., 2006, 2009; Diener et al., 2010; Granger et al., 2011; Patel et al., 2011) are systematically lower compared to the rates of ICH of non-inception and inception observational studies (Landefeld and Goldman, 1989; Fihn et al., 1993; van der Meer et al., 1993; Steffensen et al., 1997; Beyth et al., 1998; Go et al., 2003; Hylek et al., 2007; Poli et al., 2009; Figure 2). A recent observational inception cohort study of patients treated with oral anticoagulation (of whom a quarter had a previous history of stroke) reported a 2.5% (95% CI 1.1–4.7%) risk of ICH at 1 year (Hylek et al., 2007). Because oral anticoagulation-related ICH is so often fatal or disabling, even a small increase in the absolute risk of ICH of only 1% per year could potentially outweigh the benefit of oral anticoagulant treatment (Gustafsson et al., 1992), and tip the balance in favor of an alternative treatment.

### New oral anticoagulants

There has been an intense interest in recently developed oral anticoagulants that are equally efficacious but overcome the well-known limitations of warfarin, including numerous interactions with other drugs, the need for regular blood monitoring and dose adjustments and also have lower risks of intracranial bleeding (Ahrens et al., 2011; Mega, 2011; Lip, 2012). The new oral anticoagulants fall into two major categories: (a) the oral direct thrombin inhibitors (e.g., dabigatran) and (b) the oral factor Xa inhibitors (the – xabans, including rivaroxaban and apixaban). Of these agents, dabigatran was recently approved for use for stroke prevention in patients with AF, while rivaroxaban has been approved by the FDA and the European Union and apixaban is in advanced phase of clinical development.

Although the reduced risk of ICH reported with the use of newer anticoagulants is potentially a major advance in safety, some factors in the study designs merit further consideration to determine whether this reduced risk will fully translate to the real world practice of secondary stroke prevention. The landmark phase III clinical trials for these new agents, the RE-LY (dabigatran; Connolly et al., 2009), AVERROES (apixaban; Connolly et al., 2011), ARISTOTLE (apixaban; Granger et al., 2011), and ROCKET-AF (rivaroxaban; Patel et al., 2011) trials, are also subjected to similar criticisms and debates over their designs and study populations as discussed for warfarin RCTs (Ahrens et al., 2011). RE-LY (Randomized Evaluation of Long-term Anticoagulation Therapy) study was a non-inferiority trial of dabigatran versus warfarin (unlike earlier trials that established the superiority of warfarin over placebo); while the ICH rate was around 50–70% lower compared to warfarin, it is worth remembering that 50% of the participants were already taking warfarin (“warfarin survivors”) and patients with a recent ischemic stroke or TIA (within 14 days of randomization) were excluded (Connolly et al., 2009). These factors might have had an effect on the lower risk of anticoagulation-associated ICH observed.

In a predefined subgroup analysis of patients who had a history of stroke or TIA before enrollment in the RE-LY trial (3623/18113, i.e., 20% of the patients), there was also significant reduction in ICH with dabigatran (110 and 150 mg twice daily) compared with warfarin (150 mg RR 0.27, 95% CI: 0.10–0.72; 110 mg RR 0.11, 95% CI: 0.03–0.47; Diener et al., 2010). However, these patients with a previous history of ischemic stroke or TIA were slightly younger [mean age (SD): 70.2 (9.4) vs. 71.7 (8.4), *p* < 0.0001; 70.8 (10.1) vs. 71.7 (8.5), *p* = 0.008; 70.4 (9.5) vs. 71.9 (8.3), *p* < 0.0001, for 110 mg dabigatran, 150 mg dabigatran and warfarin respectively] and more likely to have received warfarin therapy before enrollment than those without a previous history of stroke or TIA (51.4% vs. 43·9%, *p* < 0·0001; 51.3% vs. 44%, *p* < 0·0001; 52.7% vs. 45.8%, *p* < 0·0001, for 110 mg dabigatran, 150 mg dabigatran and warfarin respectively; Diener et al., 2010; Lane and Lip, 2010). Similarly, the relative treatment effects (efficacy and safety) of rivaroxaban (Hankey et al., 2012) and apixaban (Easton et al., 2012) compared with warfarin were also consistent in subgroup analyses between patients who had a previous stroke or TIA and those who had no previous stroke or TIA.

Apart from potential differences in patient characteristics, the exact mechanisms for the lower rate of ICH with dabigatran and other new oral anticoagulants compared to warfarin are not yet known, but might be related to more stable anticoagulation. Indeed in the RE-LY study, an individual patient level analysis showed that those with optimal INR control (time of INR within therapeutic range: 64%) on warfarin had similar rates of hemorrhage to dabigatran. Recently, Hart et al. (2012) retrospectively analyzed the characteristics of 154 intracranial hemorrhages (46% intracerebral, 45% subdural, and 8% subarachnoid) in 18113 participants of the RE-LY trial. They found that the clinical spectrum of intracranial hemorrhages was similar for patients given warfarin and dabigatran, but with lower absolute rates of all sites of intracranial hemorrhage and fatal intracranial hemorrhages with dabigatran. Here we focus on spontaneous ICH in this sub-analysis. Independent predictors of spontaneous ICH (*n* = 63) overall were: assignment to warfarin (RR: 4.1; *p* < 0.001), previous ischemic stroke/TIA (RR: 2.7; *p* < 0.001), aspirin use (RR: 1.8; *p* < 0.02), and age (RR: 1.04 per year; *p* < 0.02; Hart et al., 2012). Aspirin use and previous ischemic stroke/TIA predicted ICH in 42 warfarin-assigned patients (i.e., 0.36% per year), but there were no significant predictors for the 21 events in patients taking dabigatran (i.e., 0.09% per year). Of note, spontaneous ICH occurred in more warfarin-treated patients with prior ischemic stroke/TIA than in patients without; however, in patients taking dabigatran, there was no significant difference (Hart et al., 2012). It could be argued that patients in the warfarin groups perhaps had a higher prevalence of bleeding-prone microangiopathies, hence the difference. Importantly, the mortality associated with spontaneous ICH averaged 52%, with no significant differences between treatment arms (Hart et al., 2012). Another interesting point is that patients with hemorrhagic transformation of their infarcts were excluded from this evaluation (Hart et al., 2012). Another recent sub-analysis of the RE-LY trial assessed the risk of bleeding with dabigatran compared with warfarin in older and younger patients with AF (Eikelboom et al., 2011): they reported that the risk of bleeding is age dependent, being higher in patients ≥75 years of age (especially for extracranial bleeding). Oldgren et al. (2011) also reported that higher CHADS2 scores were associated with increased risks for stroke or systemic embolism, bleeding, and death in patients with AF receiving oral anticoagulants in the RE-LY.

With the availability of these new oral anticoagulants (Diener et al., 2010) it is likely that even more acute cardioembolic stroke patients will be using oral anticoagulation for secondary stroke prevention. Although bleeding risks may be lower, therapeutic reversal options remain limited. At present, there are few outcome data on these agents outside RCTs (Harper et al., 2012), so natural history studies and better understanding of the mechanisms and risk factors of anticoagulation-related ICH will be very important to allow clinicians to make informed decisions.

## MRI Predictors of Anticoagulation-Associated ICH

### Cerebral small vessel disease, CAA, and cerebral microbleeds

Because oral anticoagulant-associated ICH is associated with increased age and previous stroke, and often occurs with anticoagulation intensity within the therapeutic range (Rosand et al., 2004), it is likely that at least some of the risk is related to individual patient factors: one hypothesis compatible with available data is that the risk of ICH is increased by an age-related disorder of small brain blood vessels. Anticoagulant use *per se* should not cause ICH if cerebral vessels are intact, but the presence of microangiopathy, rendering small vessels brittle and fragile, is a plausible causal or aggravating factor for such hemorrhage. Indeed, some risk stratification scores (e.g., HAS-BLED; Pisters et al., 2010) includes clinical elements that may correlate with small vessel disease (e.g., age and hypertension).

**Figure 3:**
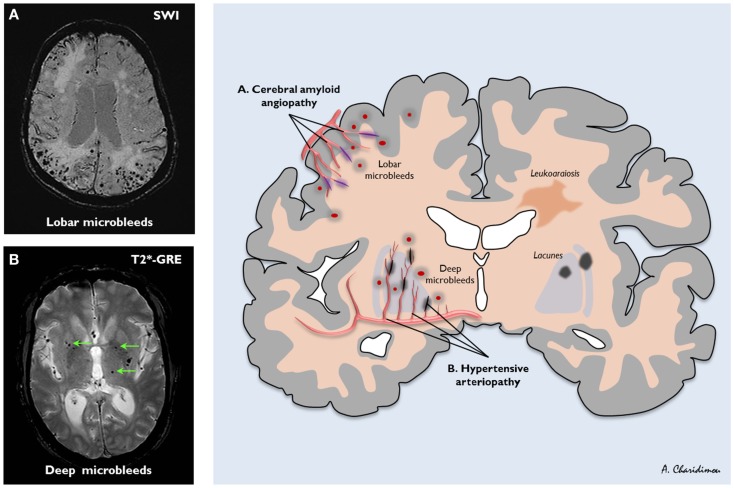
**The distribution of sporadic small vessel disease in the brain and the topography of cerebral microbleeds (CMBs)**. **(A)** Cerebral amyloid angiopathy (CAA) preferentially affects the small arteries and arterioles of the cerebral cortex and gray–white matter junction by the deposition of amyloid-β in the vessel walls (purple); **(B)** hypertensive arteriopathy typically affects small deep arterial perforators (black). CMBs are a marker for the severity and type of small vessel disease; their anatomic distribution is meant to reflect the underlying pathological vessel damage. Hence, CMBs (dark, rounded lesions) located in cortical-subcortical regions are presumably caused by CAA **(A)**, whereas CMBs located in deep brain regions mainly result from hypertensive arteriopathy **(B)**. **(A)** is an axial susceptibility-weighted imaging (SWI) which is currently the most sensitive means to image CMBs. **(B)** is an axial T2*-weighted gradient-recalled echo (T2*-GRE) MRI.

Cerebral small vessel disease is one of the most prevalent brain conditions described, especially as people live longer (Greenberg, 2006; Pantoni, 2010). The common sporadic forms are: (a) hypertensive arteriopathy (including lipohyalinosis and arteriolosclerosis), which typically affects the small perforating end-arteries of the deep gray nuclei and deep white matter, and as the name implies is related to hypertension and other traditional cardiovascular risk factors (Pantoni, 2010); and (b) CAA, a common age-related condition characterized by the progressive deposition of amyloid-β in the media and adventitia of small arteries, arterioles, and capillaries in the cerebral cortex, overlying leptomeninges, and gray–white matter junction (Charidimou et al., 2012; Figure 3). The rupture of small arteries affected by these two disease processes underlies the majority of ICHs (>75%) in the elderly, classified as spontaneous ICH (sometimes also termed primary or non-traumatic).

**Figure 4:**
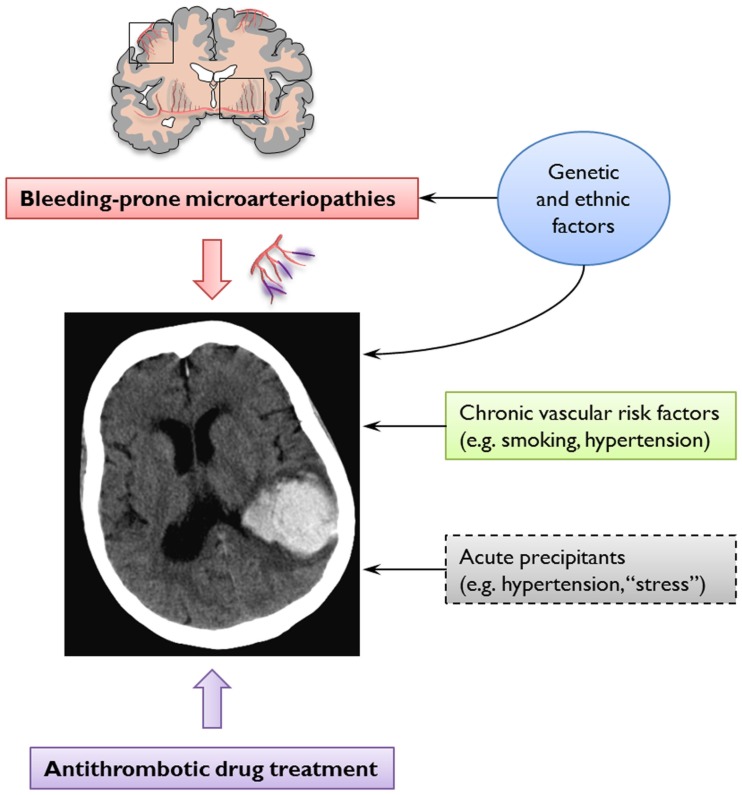
**The pathogenesis of spontaneous and anticoagulation-associated intracerebral hemorrhage (ICH) involves interaction between an underlying bleeding-prone small vessel disease (e.g., cerebral amyloid angiopathy) and the use of oral anticoagulation treatments**. This dynamic interplay is modified at various levels by genetic and ethnic factors and cardiovascular risk factors. Acute trigger factors for example, sudden increases in blood pressure or minor trauma may cause the rupture of these abnormally weak vessels. Anticoagulation may promote ICH by allowing an otherwise innocuous minor and self-limiting vessel leak to expand into a life-threatening hematoma.

Cerebral amyloid angiopathy is most often recognized in life by symptomatic, spontaneous, lobar ICH in elderly patients (Charidimou et al., 2012). Evidence supporting a link between CAA and anticoagulation-associated ICH includes the demonstration of CAA in 7 of 11 lobar ICHs occurring on warfarin in the largest consecutive pathological series reported (Rosand et al., 2000). In addition, the apolipoprotein E e2 allele, a known genetic risk factor of CAA-related lobar ICH (Nicoll et al., 1997; Biffi et al., 2010b), is more common in warfarin-associated ICH than in control patients on warfarin without ICH, supporting a role for CAA (Rosand et al., 2000). There are also individual cases of ICH following anticoagulation or coronary thrombolysis, which demonstrated advanced CAA on autopsy (Melo et al., 1993; McCarron and Nicoll, 2004). However, the mechanisms of spontaneous and anticoagulation-associated ICH are complex and involve a dynamic interplay between underlying bleeding-prone small vessel diseases, genetic factors, cardiovascular risk factors, and the use of oral anticoagulation treatments (Figure 4).

Modern MRI allows an unprecedented ability to identify cerebral small vessel disease *in vivo*. Leukoaraiosis has been recognized for many years as a characteristic MRI manifestation of small vessel disease. Some studies suggest that the presence of leukoaraiosis is associated with an increased the risk of oral anticoagulant-associated ICH (Gorter, 1999; Smith et al., 2002). However, the pathological substrates of leukoaraiosis are heterogeneous in nature and severity (ranging from myelin loss, axon loss, and mild gliosis, to microinfarction and dilation of perivascular spaces) and hence leukoaraiosis is not a very specific marker of underlying bleeding-prone microangiopathy (Gouw et al., 2010; Schmidt et al., 2011). The introduction of blood-sensitive MRI sequences, including T2*-GRE and susceptibility-weighted imaging (SWI) has enabled the accurate detection of CMBs (defined radiologically as small, rounded, homogeneous, hypointense lesions, not seen with conventional spin echo sequences) as a new imaging marker of small vessel disease (Greenberg et al., 2009). Histopathological correlation to date has shown that radiologically defined CMBs are quite specific for small collections of blood-breakdown products (in particular, hemosiderin-laded macrophages), adjacent to small vessels mainly affected by hypertensive arteriopathy or CAA (Fazekas et al., 1999; Shoamanesh et al., 2011; Werring, 2011). CMBs are thus unique among current MRI manifestations of small vessel disease, in that they seem to provide direct evidence of blood leakage from pathologically fragile small vessels (Charidimou and Werring, 2011).

Several lines of evidence show that CMB distribution in the brain reflects the underlying small vessel disease: strictly lobar (cortical-subcortical) CMBs are characteristic of CAA (allowing the diagnosis of CAA during life using the Boston criteria (Knudsen et al., 2001), whilst deep CMBs likely reflect hypertensive arteriopathy (Figure 3; Greenberg et al., 2009; Pantoni, 2010; Charidimou and Werring, 2011). If the risk of antithrombotic-related ICH is related to the underlying arteriopathy type, CMBs may be of particular value.

### How may CMBs relate to anticoagulation-associated ICH risk?

The plausible mechanism by which CMBs may be linked to anticoagulation-related ICH depends on the following postulates:

(1) CMBs reflect areas of bleeding from cerebral small vessels.
(2) CMBs are common in the populations likely to be exposed to anticoagulant drugs (Ueno et al., 2008).
(3) CMBs develop dynamically over time in a significant proportion of patients.
(4) CMBs that arise are usually “sealed off” by hemostatic factors or surrounding tissues, thus not causing obvious clinical symptoms.
(5) In the presence of anticoagulation, some CMBs are not effectively limited by these mechanisms, and may develop into a serious symptomatic ICH.

Here we briefly consider the evidence for each of these statements.

The available evidence suggests that CMBs are mostly due to previous small areas of blood leakage from damaged small vessels (Fazekas et al., 1999; Schrag et al., 2009; Shoamanesh et al., 2011; Werring, 2011) and are common in cerebrovascular disease and normal aging (Cordonnier et al., 2007). CMBs are increasingly detected in patients with stroke: there are more prevalent in patients with recurrent stroke than in patients with first-ever stroke, indicating that they are associated with the progression of small vessel cerebrovascular disease (Cordonnier et al., 2007) CMBs are also very common in the general elderly population and their prevalence increases with age (Sveinbjornsdottir et al., 2008; Poels et al., 2010). In the Rotterdam scan study, which used an optimized SWI sequence, the prevalence of CMBs was around 40% in participants over 80 years old (Poels et al., 2010). In this and other population-based studies lobar CMBs (suggesting sub-clinical CAA) were detected in up to about 25% healthy elderly individuals (Sveinbjornsdottir et al., 2008; Mesker et al., 2011), who are at high risk of AF and ischemic stroke.

Recent studies show that CMBs accumulate over time and are related to baseline CMBs in population-based healthy elderly and hospital-based stroke and memory clinic cohorts (Goos et al., 2010; Gregoire et al., 2010a; Lee et al., 2011; Poels et al., 2011). Moreover, Jeon et al. (2009) demonstrated the rapid evolving nature of CMBs even in the acute phase (days) of ischemic stroke. This study showed the rapid appearance of one or more CMBs in 12.7% of patients (*n* = 237) who underwent serial T2*-GRE at presentation and after a median of 4 days. The presence of CMBs at baseline and severe small vessel disease were independent predictors of new CMBs. Anticoagulation is hypothesized to promote ICH by allowing an otherwise innocuous minor and self-limiting vessel leak (e.g., a CMB) to expand into a life-threatening hematoma (Hart et al., 1995), especially if the leaking vessel is damaged by advanced small vessel disease (e.g., CAA). It is hypothesized that CMBs are usually “sealed off” and limited by hemostatic mechanisms and surrounding tissue, but anticoagulants, by impairing hemostatic mechanisms, increase the likelihood of a CMB enlarging into a “macrobleed.”

### What evidence is available on the role of CMBs as predictors of ICH risk after ischemic stroke?

Available evidence on the relationship of CMBs with spontaneous and anticoagulation-associated ICH comes from three types of studies: (a) cross-sectional case-control and case-case comparisons; (b) prospective studies; and (c) systematic reviews and meta-analyses.

#### Case-control and case-case studies

A numbers of studies have investigated the prevalence of CMBs in relation to antithrombotic use (Orken et al., 2009; Vernooij et al., 2009), in antithrombotic users with ICH *versus* antithrombotic users without ICH (Rosand et al., 2000; Ueno et al., 2008), and antithrombotic-associated ICH *versus* non-antithrombotic-associated ICH (Lee et al., 2009). Data from the Rotterdam scan study showed that CMBs were more prevalent among users of platelet aggregation inhibitors (adjusted odds ratio: 1.71; 95% CI: 1.21–2.41), compared with non-users of antithrombotic drugs (Vernooij et al., 2009). In a case–control study comparing 24 patients with warfarin-associated ICH with 48 warfarin users without ICH, the frequency and the number of CMB were much higher in the ICH group (79.2% vs. 22.9%, *p* < 0.001; Lee et al., 2009). Furthermore, increased prothrombin time and the presence of CMBs were both independent predictors of ICH (Lee et al., 2009). In another case-control study, lobar CMBs (suggesting possible CAA) were found to be a risk factor for aspirin-related ICH (Gregoire et al., 2010b), in line with the results of a previous report (Wong et al., 2003). A meta-analysis of published and unpublished data on stroke patient cohorts treated with antithrombotic drugs attempted to systematically bring together the available evidence (Lovelock et al., 2010). In a pooled analysis of 1460 patients with ICH and 3817 patients with ischemic stroke or TIA, case-case comparisons showed that CMBs are more common in warfarin-related ICH than “spontaneous” ICH (OR: 2.7; 95% CI: 1.6–4.4; *p* < 0.001; Lovelock et al., 2010). These results provide indirect evidence that warfarin could indeed be harmful in patients with CMBs, but there was no standardization of imaging data or CMB rating, and data could not be analyzed at an individual patient level for CMB number or location. Case-control and case-case comparisons are useful to tackle rare outcomes, but the choice of control group is critical to avoid biases and their findings cannot clearly show causative relationships.

#### Prospective cohort studies

There are limited prospective studies of CMBs which include ischemic stroke patients (Fan et al., 2003; Soo et al., 2008; Thijs et al., 2010), ICH patients (Greenberg et al., 2004; Jeon et al., 2007) or healthy elderly individuals (Bokura et al., 2011). Table 3 summarizes some key aspects of the main prospective cohort studies that have assessed the risk of ICH and CMBs in patients after ischemic stroke or TIA. Many of these studies were not specifically designed to answer the question of CMBs and antithrombotic ICH risk, and they also suffer from methodological limitations including: (a) small sample size; (b) varied CMB imaging parameters between studies; and (c) lack of analysis of ICH risk in relation to CMB number and anatomical location.

**Table 3:** **Characteristics, study design and methodological aspects of the main prospective cohort studies which have assessed the risk of future intracerebral hemorrhage in relation to the presence of cerebral microbleeds (CMBs)**.

Study	Country	Participants		Antithrombotic users		T2*-GRE MRI parameters			CMBs prevalence	FU time
		*n*	Mean age, y	Antiplatelet users	Warfarin users	Field strength (Tesla)	Echo time (ms)	Section thickness (mm)
Thijs et al. (2010)	Belgium	487	72	32%		1/1.5/3	35/26/16	7	26.5%	2.2 years (median)
Orken et al. (2009)	Turkey	141	65.8	–	100%	1.5	15	5	22%	3.94 years (mean)
Soo et al. (2008)	China	908	–	92.5%	4.3%	1.5	30	5	27.8%	26.6 months (mean)
Naka et al. (2006)	Japan	183	–	–	–	1	26	5	29%	1.54 years
Boulanger et al. (2006)	Canada	236	–	23.7%	–	3	20	5	19.1%	14 month (median)
Fan et al. (2003)	China	121	68	80%	5.8%	1.5	30	5	35.5%	27.5 months (mean)

In a study of 121 consecutive patients with ischemic stroke, followed up for 27 ± 12 months, four patients with CMBs (9.3%) and one patient without (1.3%) had ICH (*p* = 0.05; Fan et al., 2003). Two of the hematomas occurred in the site where CMBs were found at baseline (Fan et al., 2003). The largest prospective cohort of CMBs and antithrombotic use included 908 patients with ischemic stroke, treated with a single antithrombotic agent (93% with aspirin) with mean follow-up of 26 months (Soo et al., 2008). Both age and CMBs were found to be independent predictors of subsequent ICH. During follow-up, it was found that the risk of ICH increased significantly with the burden of CMBs: 0.6% in patients with no CMB, 1.9% in patients with one CMB, 4.6% in patients with two to four CMBs and 7.6% in patients with five or more CMBs (*p* < 0.001; Soo et al., 2008). In this study, overall, 15/908 (1.7%) patients suffered ICH at an average follow-up time of 27 months; of patients with CMBs, 4.4% developed ICH, whilst 0.6% of patients without CMBs developed ICH (Soo et al., 2008). Thus the relative risk of the finding of CMBs for subsequent ICH is 7.3 (4.4/0.6). However, this was an Asian cohort, and their findings may not be generalizable to other populations: CMBs were detected in 27.8% of the patients and were most commonly observed in basal ganglia and thalamus, suggesting that hypertensive arteriopathy was the predominant small vessel pathology.

It is worth remembering that the presence of CMBs is associated with an increased risk of future ischemic stroke as well as ICH. For example, a recently published prospective follow-up study (median 2.2 years) of a European cohort of 487 hospitalized patients with a TIA or ischemic stroke, found that patients with CMBs had a higher risk of developing new ischemic stroke rather than ICH (Thijs et al., 2010): during follow-up, only two patients developed ICH, compared to 32 patients who developed recurrent ischemic stroke, and three with undetermined stroke. Only strictly lobar CMBs (or combined with deep microbleeds) had an independent effect on the risk of recurrent stroke (*p* = 0.018; Thijs et al., 2010).

In pooled follow-up data from 768 antithrombotic users including the Oxford Vascular Study (OxVASC) cohort (ICH, ischemic stroke, and TIA patients), CMBs at baseline were associated with higher risk of ICH (OR: 12.1, 95% CI: 3.4–42.5, *p* < 0.001; Lovelock et al., 2010).

**Figure 5:**
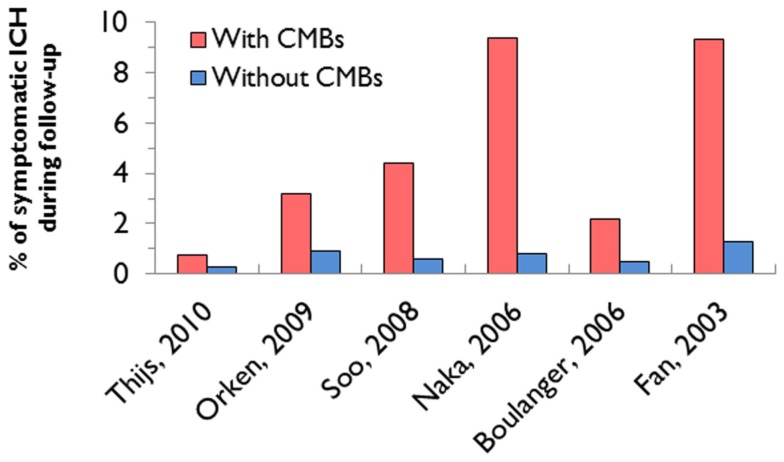
**Incidence of intracerebral hemorrhage in relation to the presence of cerebral microbleeds (CMBs) in the main prospective cohort studies which have assessed this risk in patients with ischemic stroke or TIA (Table 3)**.

Taken together, prospective studies after ischemic stroke suggest a substantially increased hazard of ICH in the presence of CMBs (Figure 5), but there is considerable heterogeneity in the cohorts studied. It is important to note that many of the studies to date have not used standardized rating scales for CMBs, and have not reported the ICH risk in relation to CMB anatomical location. The risk of recurrent bleeding after symptomatic ICH seems to be higher for lobar ICH (often presumed due to CAA; Vinters, 1987; Passero et al., 1995). Lobar CMBs may thus be a stronger risk factor for antithrombotic-associated ICH than deep CMBs, but data are lacking. A recent single center study of 104 survivors of “spontaneous” ICH attributed to CAA demonstrated that recurrent ICH was associated with baseline lobar CMBs (hazard ratio: 2.93 for 2–4 CMBs and 4.12 for >5 CMBs; Biffi et al., 2010a).

No large prospective studies of CMBs in ischemic stroke cohorts treated with anticoagulants for AF have been completed to date, although this population reflects a common therapeutic dilemma in clinical practice. Further prospective data will assist the development of a reliable risk model, incorporating the most promising neuroimaging markers of bleeding risk (including CMBs), to aid anticoagulation decisions in stroke populations who may have the highest risk of ICH. One large prospective European multicenter MRI study is currently underway in the UK and is briefly described in the next section.

## CROMIS-2: Aims and Study Design

The CROMIS-2 (Clinical Relevance Of Microbleeds In Stroke) study^2^, aims to establish the value of CMBs (as well as other neuroimaging markers) and genetic factors in predicting symptomatic ICH following best practice oral anticoagulation to prevent recurrent ischemic stroke due to AF.

In summary, CROMIS-2 will set out to answer the following key questions:

(1) Does the presence of CMBs help predict the risk of symptomatic oral anticoagulant-associated ICH in patients who are anticoagulated following cardioembolic stroke due to AF?
(2) Do the burden (number) and distribution of CMBs at baseline influence the risk of ICH in this cohort (independently of other clinical and imaging factors)?
(3) Are CMBs associated with an increased risk of recurrent TIA, ischemic stroke or death in these patients?
(4) Can we identify new genetic, clinical, or radiological risk factors of anticoagulant-associated ICH?

These questions will be addressed by two complementary studies (their inclusion and exclusion criteria are summarized in Table 4).

**Table 4:** **CROMIS-2 inclusion and exclusion criteria**.

Inclusion criteria	Exclusion criteria
**STUDY I: CROMIS-2 (AF)**	
Adult (≥ 18y; no upper limit) patients with a clinical diagnosis of non-valvular	Any MRI contraindications
AF (verified by ECG) and intention to treat with best practice oral anticoagulants (e.g., warfarin)	Previous ischemic stroke or TIA diagnosed by treating clinician Definite contra-indication to oral anticoagulation
Previous therapeutic use of oral anticoagulation	Serious head injury (resulting to loss of consciousness)
All patients must be able to have T2*-GRE MRI before (or within 1 week) of starting oral anticoagulation
**STUDY II: CROMIS-2 (ICH)**	
Adult (≥ 18y) patients treated at participating centers with confirmed spontaneous ICH (on CT or MRI scans) with or without a history of anticoagulant use at the time of the ICH	Known underlying structural cause for ICH (e.g., arteriovenous malformation, tumor, cavernoma, intracranial aneurysm) Major head trauma (causing loss of consciousness and though to be sufficient to have caused the ICH) in previous 24 h

### Study I

CROMIS-2 (AF) is a prospective inception cohort study (*n* = 1000) of patients throughout the UK started on best practice oral anticoagulants (without prior use) for presumed cardioembolic ischemic stroke due non-valvular AF. Patients will have standardized MRI including T2*-GRE at baseline. Imaging analysis will take place at the co-ordinating center using appropriate validated rating scales for CMBs and other markers of cerebrovascular disease. Patients with be followed up for an average of 2 years. The primary outcome will be symptomatic ICH (confirmed on brain imaging); the secondary outcomes will be recurrent ischemic stroke or TIA and death of any cause.

### Study II

CROMIS-2 (ICH) is an observational and genetics study of ICH: 600 patients admitted to participating centers with spontaneous ICH (with a target of at least 300 anticoagulant-associated ICH cases) will be recruited. Clinical, imaging and genetic data from these ICH cases will be collected to build a UK-wide ICH registry for the investigation of risk factors associated with anticoagulant-related ICH compared to non-anticoagulant-related ICH. Patients will be followed up at 6 months.

### Statistical considerations

We expect the total ICH rate in our cohort to be at least 2.5% per year. The relative risk for ICH of having CMBs is not well established. If we assume a relative risk similar to the one found in the largest prospective data in an ischemic stroke cohort investigated for CMBs published to date (i.e., 7.3; Soo et al., 2008), then we would expect a rate of ICH at 2 years follow-up of 6.5% in patients with CMBs, compared with 0.9% without CMBs: this difference would be clinically important and would tip the risk-benefit judgment in favor of avoiding or reducing the intensity of oral anticoagulation, or substituting an antiplatelet agent in patients with CMBs. These estimated risks suggest that we will observe 30 events in total over a 2-year period (20 in patients with CMBs, and 10 in those without). The “rule of 10” for developing risk models suggests that this will allow us to develop a risk model with three predictor variables in total. A risk model based solely on CMBs would have a sensitivity of 67% and a specificity of 82% for predicting an ICH within 2 years. The positive predictive value would be 14%. A risk model with more variables should improve on these values.

## Conclusion

**Figure 6:**
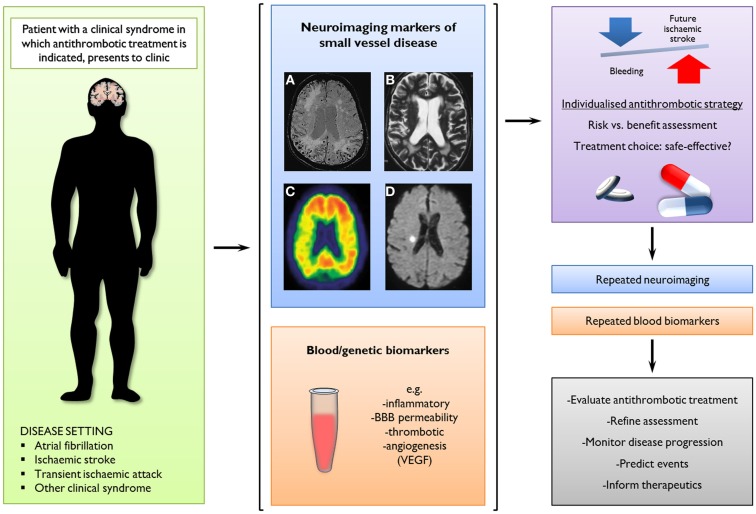
**A potential new clinical paradigm of the future role of neuroimaging markers of small vessel disease and blood/genetic biomarkers to predict intracerebral hemorrhage (ICH) by guiding stratified antithrombotic treatment decisions**. In this scenario, a combination of imaging markers could be used to assess the relative balance of risk for future ischemic stroke or ICH, informing therapeutic decisions as well as potentially evaluating treatment effects and monitoring disease progression. Neuroimaging modalities and findings with potential clinical implications include: **(A)** cerebral microbleeds on axial T2*-weighed gradient-recalled echo or susceptibility-weighted imaging; **(B)** white matter changes on axial T2-weighted MRI; **(C)** amyloid-b load on PET images using radioligands (e.g., Pittsburgh compound B); and **(D)** small acute ischemic lesions (possibly cerebral microinfarcts) on axial diffusion-weighted images.

The increasing use of antithrombotic drugs in an aging population (including anticoagulants to prevent future ischemic stroke in individuals with ischemic stroke due to AF) has led to a dramatic increase in the incidence of ICH associated with antithrombotic drug use. Several lines of evidence suggest that cerebral small vessel disease (particularly CAA) is a risk factor for this rare but devastating complication. Although RCTs suggest that the rate of symptomatic ICH in anticoagulated patients is low (particularly with the use of new alternatives to warfarin), these studies are not fully reflective of clinical practice, and are not the optimal way to investigate predictors of rare adverse events (Vandenbroucke, 2011). CMBs have emerged as a potentially powerful marker of future ICH risk, but high quality prospective studies of CMBs and ICH risk on anticoagulation are not available. Further data are urgently needed to determine how neuroimaging and other biomarkers (e.g., genetic variations) may contribute to individualized risk prediction to make anticoagulation as safe and effective as possible (Figure 6).

## Conflict of Interest Statement

The authors declare that the research was conducted in the absence of any commercial or financial relationships that could be construed as a potential conflict of interest.

## Footnotes

^1^http://www.ucl.ac.uk/cromis-2/

^2^www.ucl.ac.uk/cromis-2
